# Statistical Analysis and Prediction of Fatal Accidents in the Metallurgical Industry in China

**DOI:** 10.3390/ijerph17113790

**Published:** 2020-05-27

**Authors:** Qingwei Xu, Kaili Xu

**Affiliations:** 1 College of Information and Management Science, Henan Agricultural University, Zhengzhou 450046, China; 2 Key Laboratory of Ministry of Education on Safe Mining of Deep Metal Mines, School of Resources and Civil Engineering, Northeastern University, Shenyang 110819, China; xukaili@mail.neu.edu.cn

**Keywords:** metallurgical industry, fatal accidents, grey interval predicting method, GM(1,1) model, bow tie model

## Abstract

The metallurgical industry is a significant component of the national economy. The main purpose of this study was to establish a composite risk analysis method for fatal accidents in the metallurgical industry. We collected 152 fatal accidents in the Chinese metallurgical industry from 2001 to 2018, including 141 major accidents, 10 severe accidents, and 1 extraordinarily severe accident, together resulting in 731 deaths. Different from traffic or chemical industry accidents, most of the accidents in the metallurgical industry are poisoning and asphyxiation accidents, which account for 40% of the total number of fatal accidents. As the original statistical data of fatal accidents in the metallurgical industry have irregular fluctuations, the traditional prediction methods, such as linear or quadratic regression models, cannot be used to predict their future characteristics. To overcome this issue, the grey interval predicting method and the GM(1,1) model of grey system theory are introduced to predict the future characteristics of fatal accidents in the metallurgical industry. Different from a fault tree analysis or event tree analysis, the bow tie model integrates the basic causes, possible consequences, and corresponding safety measures of an accident in a transparent diagram. In this study, the bow tie model was used to identify the causes and consequences of fatal accidents in the metallurgical industry; then, corresponding safety measures were adopted to reduce the risk.

## 1. Introduction

With the rapid economic development in China, people’s living standards continue to improve [1,2,3]. The metallurgical industry makes products that serve as the foundation of modern industry, and the success of this industry is linked to all aspects of the national economy [4,5]. The metallurgical industry includes the smelting and pressing of ferrous metals and the smelting and pressing of non-ferrous metals [6,7,8]. Ferrous metals include iron, chrome, manganese, and their alloys, while the other metals are referred to as nonferrous metals [9,10,11].

According to Dr. Edwin Basson, director-general of the World Steel Association, the steel industry has made outstanding contributions to promoting global economic growth and employment [12]. In terms of value-added, every dollar of value-added that is created by the steel industry will drive other global economic sectors to create an added value of $2.5 [12]. In terms of employment, every two employment positions that are created by the steel industry can drive the global steel supply chain to create thirteen employment positions [12]. The contributions of the entire metallurgical industry to global development are much more than these. While benefiting global development, the metallurgical industry is also dangerous, and fatal accidents can occur [13,14,15], presenting high costs to businesses and society. For example, a thermal injury accident occurred at Qinghe Special Steel Co. Ltd. on 19 August 2007, which caused 32 deaths and six injuries, and a poisoning and asphyxiation accident occurred at Puyang Iron and Steel Co. Ltd. on 4 January 2010, causing 21 deaths and 9 injuries (State Administration of Work Safety (SAWS)) [16]. The metallurgical industry is a high-risk industry; statistical analysis has been performed for risks in other dangerous fields, such as coal mining [17,18,19], the transportation industry [20,21,22] and the chemical industry [23,24,25]. Zhang et al. analyzed the patterns and characteristics of 126 gas explosion accidents in Chinese coal mines [18]. Sze and Wong reported the injury, demographic, crash environmental, geometric and traffic characteristics of 73,746 pedestrian casualties that were involved in traffic crashes [20]. Shin described the characteristics of 147 major chemical industry accidents that occurred in Korea [23]. Despite the serious consequences of fatal accidents in the metallurgical industry, few statistical analysis reports of fatal accidents in this industry have been performed. Li et al. counted the accidents in large scale iron and steel enterprises in 2010, 2011, and 2012 [26], but there are deficiencies in their study. First, the scope of the statistics is only large scale iron and steel enterprises, which cannot represent all the enterprises in the metallurgical industry. Second, the statistical period was only three years, so the results based on these data may be inaccurate. In addition, Berhan investigated the prevalence of workplace accidents and the associated factors in the iron, steel and metal manufacturing industries and collected primary data using a questionnaire method from production workers [27]; however, the results may be seriously affected by the subjectivity of the employees. Due to the important role of the metallurgical industry in global development, it is critical to perform a statistical analysis of the characteristics of the fatal accidents in the metallurgical industry. Importantly, this type of analysis is required to design appropriate safety measures to ensure safe production in the metallurgical industry.

Once the accident statistics in the metallurgical industry have been collected, not only should the patterns and characteristics of the accidents be analyzed, but the future characteristics of the metallurgical industry must also be predicted to adopt the corresponding safety measures. The widely used prediction techniques include grey system theory [28,29,30], BP neural network [31,32,33], linear regression analysis [34,35,36], and nonlinear regression analysis [37,38]. Wang et al. predicted the urban heat supply based on the grey system theory [29]. Xu et al. proposed a new method for determining the number of hidden neurons in a network according to the mean square error of training samples and predicted the sand casting performance parameter based on the BP neural network method [32]. Xu et al. predicted the maximal water bursting discharge from the coal seam floor based on multiple nonlinear regression analysis [38]. For the situation where irregular fluctuations occur in the original data, it is usually impossible to find a suitable model to describe the variation tendency, so it is impossible to accurately predict future characteristics. For this situation, we could consider predicting the variation range of the dependent variable. Fortunately, the interval predicting method of the grey system theory can deal with this issue [39,40,41]. In addition, although linear or nonlinear regression analysis can achieve better prediction results for some issues, these techniques are powerless for predicting catastrophes. However, the GM(1,1) model of the grey system theory can be used for catastrophe prediction [42,43,44]. Therefore, the grey system theory is introduced to predict the future characteristics of fatal accidents in the metallurgical industry.

If the future characteristics of the accidents in the metallurgical industry were serious, specific safety measures should be adopted to limit the impacts of dangerous and potentially harmful factors as a strategy to eliminate the potential for accidents. The bow tie model is a widely used risk analysis tool, different from fault tree analysis [45,46,47] or event tree analysis [48], the bow tie model integrates the basic causes, possible consequences, and corresponding safety measures of an accident in a transparent diagram [49,50,51]. Xu et al. used the bow tie model to conduct a detailed analysis of critical indicators in a petrochemical enterprise to prevent accidents [49]. In this study, the bow tie model was used to identify the causes and consequences of accidents in the metallurgical industry; then, corresponding safety measures were adopted to reduce the risk of accidents.

The main purpose of this study was to establish a composite risk analysis method for fatal accidents in the metallurgical industry. The accident characteristics of fatal accidents in the metallurgical industry were analyzed based on statistical data. Then, the future characteristics of fatal accidents in the metallurgical industry were predicted by the grey system theory. Finally, the bow tie model was used to identify the causes and consequences of fatal accidents in the metallurgical industry, and corresponding safety measures were adopted to reduce the risk of accidents.

## 2. Methods

### 2.1. Production Accident Statistics

According to the guidelines for the reporting, investigation, and handling of production accidents, production accidents can be classified based on casualties or direct economic loss [18], as shown in Table 1.

If the numerical value of deaths, serious injuries or/and property loss is larger than the severe accident category range, then the accident grade is extraordinarily severe. The direct economic loss resulting from a production accident is difficult to estimate, and this information is not typically included in accident investigation reports. The accident level is usually determined based on the number of deaths and serious injuries.

There are many ordinary accidents, and sometimes the available website records are not complete. In addition, some businesses may not publicize information about ordinary, or less severe accidents to protect their corporate image. Therefore, this study focused on fatal accidents, which including major, severe, and extraordinarily severe accidents, with the idea that there is better reporting of these accidents. 

This study examined a total of 152 accidents in the metallurgical industry in China from 2001 to 2018, which included 141 major accidents, 10 severe accidents, and 1 extraordinarily severe accident. The statistical data on production accidents were obtained from the accident inquiry system of the government website of the State Administration of Work Safety (SAWS) and the accident investigation reports of the local government website [16]. The statistical data for 2018 were only available until 30 June; after that, no additional data were available on the SAWS website. All the statistical data for the studied accidents are provided as Supplementary Materials.

### 2.2. GM(1,1) Model

Although linear regression analysis [34,35,36] and nonlinear regression analysis [37,38] can achieve better prediction results for some issues, they are unable to predict catastrophic events. However, the GM(1,1) model of the grey system theory can be used for catastrophe prediction [42,43,44]. The GM(1,1) model-based catastrophe prediction has been applied in many situations, such as the seasonal disaster prediction of flood [52], artificial muscle tremor behavior prediction [53], and epidemic peaks prediction for typhoid and paratyphoid fever [54]. The task of GM(1,1) model-based catastrophe prediction is to determine the moment when the next or several outliers will appear so that people can prepare in advance and take countermeasures.

Assume that *X*^(0)^ = (*x*^(0)^(1), *x*^(0)^(2), …, *x*^(0)^(*n*)) is the original data series, and *X*^(1)^ = (*x*^(1)^(1), *x*^(1)^(2), …, *x*^(1)^(*n*)) is the first-order accumulating data series, where *x*^(1)^(*k*) can be calculated based on the first-order accumulating generation operator (1-AGO) as follows:

(1) $$x^{\left(1\right)}(k)=\sum_{i=1}^{k}x^{\left(0\right)}(i),k=1,2,\cdots ,n$$

where *x*^(0)^(*k*) indicates the original data, and *x*^(1)^(*k*) indicates the 1-AGO data.

Assume that the matrix *X*^(1)^ accords with the exponential change law, and the whitenization equation of the GM(1,1) model is shown as follows:

(2) $$\frac{dx^{\left(1\right)}}{dt}+ax^{\left(1\right)}=b$$

where *t* indicates the time; *a* indicates the developing coefficient; *b* indicates the grey input.

Let ${\hat{x}}^{\left(1\right)}(1)=x^{\left(0\right)}(1)$ be the initial condition, solve Equation (2) and the predictive formula of *X*^(1)^ can be obtained, as shown in Equation (3) [42].

(3) $${\hat{x}}^{\left(1\right)}\left(k+1\right)=\left[x^{\left(0\right)}\left(1\right)-\frac{b}{a}\right]e^{-ak}+\frac{b}{a},\;\;\;k=0,1,2,\cdots$$

where ${\hat{x}}^{\left(1\right)}\left(k+1\right)$ indicates the predictive value of 1-AGO.

The predictive formula *X*^(0)^ of the original data series is shown in Equation (4), calculated as $x^{\left(1\right)}\left(k+1\right)-x^{\left(1\right)}\left(k\right)$.

(4) $${\hat{x}}^{\left(0\right)}\left(k+1\right)=\left(1-e^{a}\right)\left[x^{\left(0\right)}\left(1\right)-\frac{b}{a}\right]e^{-ak},\;\;\;k=1,2,3,\cdots$$

where ${\hat{x}}^{\left(0\right)}\left(k+1\right)$ indicates the predictive value of the original data series.

The developing coefficient *a* and the grey input *b* are based on the least square estimation of the GM(1,1) model, as shown in Equation (5).

(5) $$\hat{a}={\left(B^{T}B\right)}^{-1}B^{T}Y={\left(a,b\right)}^{T}$$

where the matrix *B* and matrix *Y* are as follow.

$$B=\left(\begin{array}{cc} -Z^{\left(1\right)}\left(2\right) & 1\\ -Z^{\left(1\right)}\left(3\right) & 1\\ \cdots & \cdots \\ -Z^{\left(1\right)}\left(n\right) & 1 \end{array}\right),\;\;\;Y=\left(\begin{array}{l} x^{\left(0\right)}\left(2\right)\\ x^{\left(0\right)}\left(3\right)\\ \;\;\;\cdots \\ x^{\left(0\right)}\left(n\right) \end{array}\right)$$

The background value *Z*^(1)^ is the mean series of *X*^(1)^, as calculated by the following equation:

(6) $$Z^{\left(1\right)}\left(k+1\right)=\frac{1}{2}\left[X^{\left(1\right)}\left(k+1\right)+X^{\left(1\right)}\left(k\right)\right],\;\;\;k=1,2,\cdots ,n-1$$

where *Z*^(1)^(*k* + 1) indicates the background value.

For the GM(1,1) model, a model test should be carried out before the prediction. If the given GM(1,1) model is able to pass the model test, then it can be used for predictions. The widely used model test method for the grey system is relative error testing. The main procedures of the relative error test are as follows.

The relative error of the predicted value can be obtained as follows:

(7) $$\Delta k=\frac{\left|x^{\left(0\right)}\left(k\right)-{\hat{x}}^{\left(0\right)}\left(k\right)\right|}{x^{\left(0\right)}\left(k\right)}$$

The accuracy test level is shown in Table 2.

If the relative error of the GM(1,1) model is less than 0.2, then the GM(1,1) model can be used for predicting.

### 2.3. Grey Interval Predicting Method

If the original data have irregular fluctuations, the traditional prediction methods, such as linear regression analysis [34,35,36] and quadratic regression analysis [37], cannot be used to predict their future characteristics. To overcome this issue, we consider predicting the variation range of the original data based on the grey interval predicting method [39,40,41]. Huang and Lin adopted the grey interval predicting method to predict international passenger arrivals to Taiwan [55], while Chen et al. adopted the grey interval predicting method to predict the passenger volume of the high-speed rail in Taiwan [56]. The task of grey interval predicting is to process data in accordance with the method of the grey system theory based on mastering the existing data and to understand the development results of the object in advance to take corresponding measures.

For the original data *X*^(0)^, let $\sigma_{\max}=\underset{1\leq k\leq n}{\max}\left\{x^{\left(0\right)}\left(k\right)\right\}$ and $\sigma_{\min}=\underset{1\leq k\leq n}{\min}\left\{x^{\left(0\right)}\left(k\right)\right\}$.

The upper bound function of *x*^(1)^(*k*) is shown as follows [31]:

(8) $$f_{u}\left(n+t\right)=x^{1}\left(n\right)+t\sigma_{\max}.$$

The lower bound function of *x*^(1)^(*k*) is shown as follows:

(9) $$f_{l}\left(n+t\right)=x^{1}\left(n\right)+t\sigma_{\min}.$$

The highest and lowest predictive values of *x*^(1)^(*k*) are the upper and lower bounds, respectively. The basic predictive function of *X*^(1)^(*k*) is shown as follows:

(10) $$f_{b}\left(n+t\right)=\frac{f_{u}\left(n+t\right)+f_{l}\left(n+t\right)}{2}$$

Then, the basic predictive value of original data *X*^(0)^ can be achieved as follows.

(11) $${\hat{x}}^{\left(0\right)}\left(k\right)=x^{\left(1\right)}\left(k\right)-x^{\left(1\right)}\left(k-1\right).$$

### 2.4. Bow Tie Model

**Figure 1:**
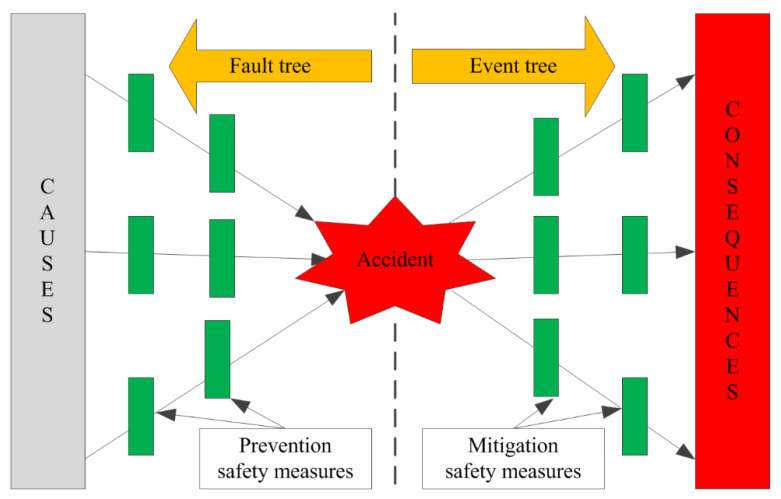
Bow tie model.

Fault tree analysis can identify the causes of an accident but cannot perform a detailed analysis of the identified risk factors [45,46,47]. Event tree analysis can perform a detailed analysis of the identified risk factors but cannot identify the causes of an accident [48]. Different from fault tree analysis [45,46,47] or event tree analysis [48], the bow tie model integrates the basic causes, possible consequences, and corresponding safety measures of an accident in a transparent diagram (Figure 1) [49,50,51]. The accident causes were indicated on the fault tree side and consequences on the event tree side. Prevention safety measures were set on the accident’s left and mitigation safety measures were set on the accident’s right. The bow tie model has been applied to many aspects of risk analysis, such as coal mine safety [50,51], petrochemical safety [49], and oxygen lance safety [57]. The task of the bow tie model is to identify the causes and consequences of an accident and then adopt corresponding safety measures to reduce the risk of an accident.

## 3. Results

### 3.1. Major Accidents of the Metallurgical Industry

**Figure 2:**
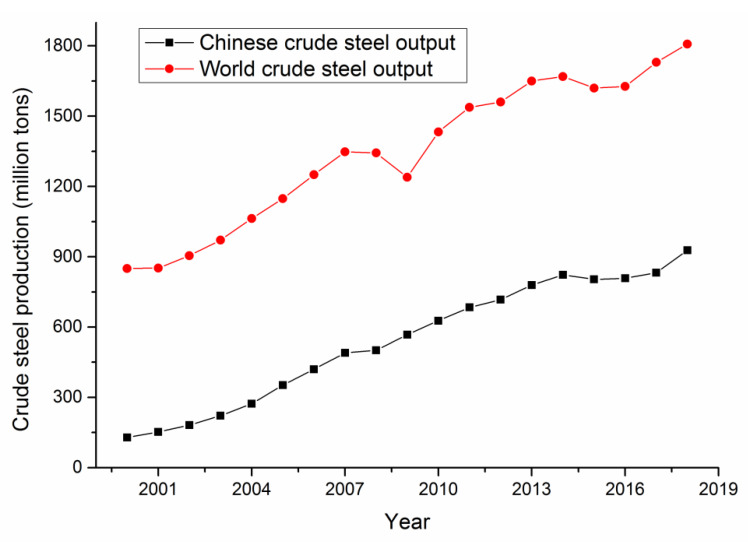
Crude steel output between 2000 and 2018.

Metallurgy is the extraction of metals or metallic compounds from minerals using various processing methods and the manufacturing of metals into materials with certain properties. Steel is the most important and widely used metallic material, and the Chinese crude steel output has been increasing since 2000 [12], as shown in Figure 2. 

As shown in Figure 2, the Chinese crude steel output remains high, although the growth of this industry slowed after 2014 in response to national economic structural adjustment policies. Chinese crude steel production has been the highest in the world for 23 consecutive years and accounts for approximately half of the world production according to the World Steel Association [12]. In 2018, the Chinese crude steel output was approximately eight times that of India, nine times that of Japan and 10 times that of the US, the countries that ranked second, third, and fourth in the world in terms of production, respectively. The crude steel output of the top nine producers after China totaled 580.7 million tons in 2018, which is 347.6 million tons less than the output of China.

However, Chinese crude steel is mostly mid- and low-end steel, and the metallurgical equipment and technology in use in China is significantly less advanced than that used in more developed countries, contributing to frequent accidents in Chinese metallurgical enterprises.

**Figure 3:**
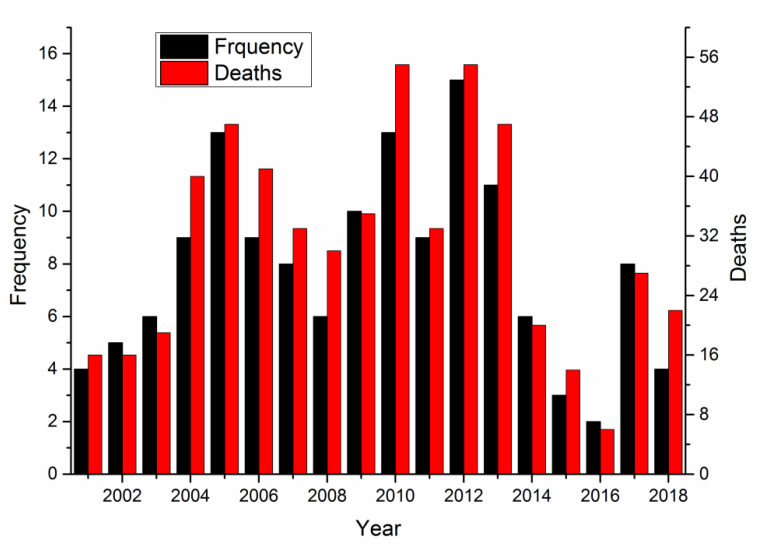
Major accidents and deaths in the metallurgical industry.

There were 152 major, severe, and extraordinarily severe metallurgical industrial accidents reported in China between 2001 and 2018, causing 731 deaths in total. The accidents occurred during regular production, maintenance, and repair activities, as well as during plant reconstruction and expansion. The major accidents that occurred between 2001 and 2018 are shown in Figure 3.

As shown in Figure 3, the numbers of major accidents and deaths show roughly saddle-type fluctuations. The most major accidents occurred in 2012, with 15 incidents, followed by 2005 and 2010 with 13. Deaths were the highest in 2010 and 2012, i.e., 55 each year, followed by 2005 and 2013, with 47 each year. Both the number of major accidents and deaths in 2016 were the lowest, with 2 and 6, respectively.

Some safe work practices recently adopted by Chinese metallurgical companies have been effective for accident prevention in individual years, but overall, safe production has not yet been achieved. The metallurgical industry includes many production processes, and a mistake at any step may lead to an accident. Some problems may also arise from insufficient production management.

### 3.2. Severe and Extraordinarily Severe Accidents of the Metallurgical Industry

The materials used in the metallurgical production processes are handled under high temperature and high-pressure conditions, and these materials are typically flammable, explosive, poisonous, and harmful. Therefore, metallurgical enterprises are prone to explosions, poisoning, and thermal injury, which can easily cause mass death and casualty. The reported severe and extraordinarily severe accidents in the Chinese metallurgical industry between 2001 and 2018 are shown in Table 3.

Table 3 describes one extraordinarily severe and ten severe accidents that occurred between 2001 and 2018, resulting in 175 deaths.

The total number of major accidents, severe accidents, and extraordinarily severe accidents in the Chinese metallurgical industry between 2001 and 2018 was 152, causing 731 deaths in total and an average of 41 deaths per year. According to Japan’s Kyodo News, the number of deaths in the Japanese metallurgical industry was 11 in 2015. This illustrates the less safe production of the Chinese metallurgical industry compared with that in other countries.

### 3.3. Accident Types of the Metallurgical Industry

**Figure 4:**
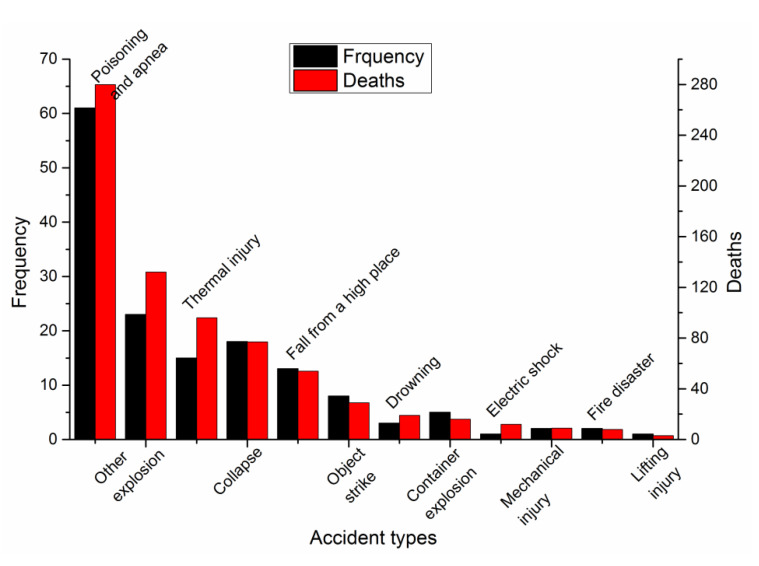
Accident type classification of reported accidents in the metallurgical industry.

According to the classification standard of casualty accidents for employees working in companies (China) [58,59], accidents can be classified as shown in Figure 4 and are explained in detail in Table 4.

As shown in Figure 4, the greatest number of accidents were poisoning and asphyxiation accidents, causing 61 cases and 280 deaths and accounting for 40% and 38% of the total number of accidents and deaths, respectively. There were 23 accidents involving a furnace, iron, or steel ladle explosion, causing 132 deaths and accounting for 15% and 18% of the total number of accidents and deaths, respectively. These two types of accidents were the main kinds of accidents and accounted for 55% and 56% of the total number of accidents and deaths, respectively.

### 3.4. Accident Occurrence Time of the Metallurgical Industry

**Figure 5:**
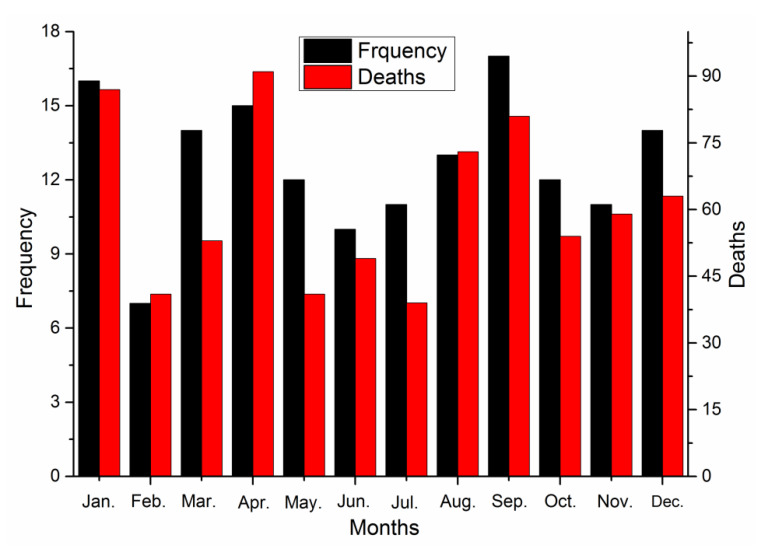
Accident occurrence time by month.

The accidents were next sorted based on the month in which they occurred, as shown in Figure 5.

As shown in Figure 5, the largest number of accidents for a single month occurred in September, with 17 events accounting for 11% of the total number of accidents. The largest number of deaths in a single month occurred in April, with 91 deaths and accounting for 12% of the total number of accidents. The second-highest number of accidents and deaths both occurred in January, with 16 accidents and 87 deaths. The immediate causes for the frequent occurrences in these three months are mainly due to employee misoperation, violating operating regulations, and the lack of a practical contingency plan. The root cause is that the employee safety training in metallurgical enterprises is insufficient, the safety awareness of employees is weak, and a good enterprise safety culture has not been formed.

The fewest accidents for a single month occurred in February, which is likely due to the Chinese New Year and the week of holiday for the Spring Festival. Overall, production is likely the lowest in February, so there were fewer accidents.

**Figure 6:**
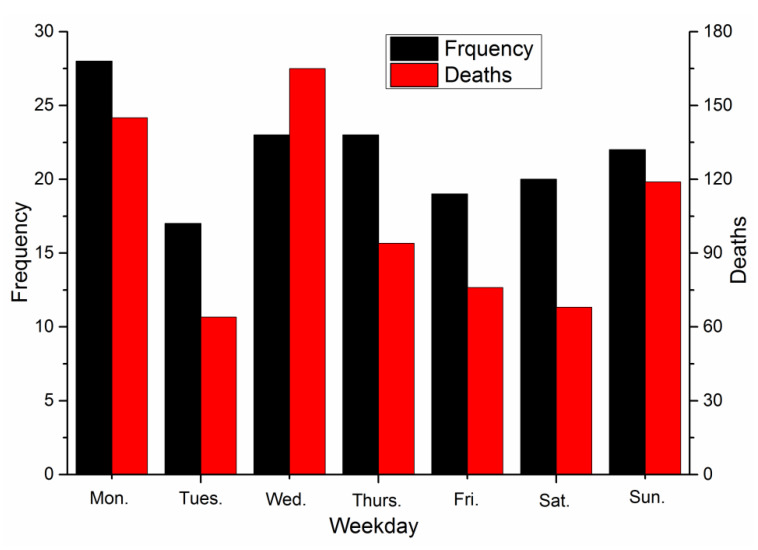
Accidents classified by the day of the week.

The accidents were next classified based on the day of the week on which they occurred, as shown in Figure 6.

As shown in Figure 6, the greatest number of accidents occurred on Monday, with 28 incidents accounting for 18% of the total number of accidents. This is likely because Monday is typically the first day of the workweek in China. The employees may not yet be adjusted to a work routine, leading to mistakes.

The three days with the highest number of deaths were Monday, Wednesday, and Sunday, with 145, 165 and 119 deaths, respectively. This correlated with the high number of severe or extraordinarily severe accidents that occurred on these three weekdays. Two severe accidents occurred on Mondays, resulting in 34 deaths; four severe accidents and one extraordinarily severe accident occurred on Wednesdays, with 86 deaths; three severe accidents occurred on Sundays, with 43 deaths.

### 3.5. Prediction of Deaths Due to Fatal Accidents by the Grey Interval Predicting Method

The original data of deaths due to fatal accidents are *X*^(0)^ = (16, 16, 19, 52, 47, 53, 85, 47, 35, 76, 45, 81, 47, 20, 24, 19, 27), and the time series of deaths of fatal accidents is *T* = (1, 2, 3, 4, 5, 6, 7, 8, 9, 10, 11, 12, 13, 14, 15, 16, 17).

The GM(1,1) model was directly established based on the original data of deaths due to fatal accidents and Equations (1)–(6), and its prediction formula is shown in Table 5. In addition, taking *T* as the independent variable and *X*^(0)^ as the dependent variable, the simple linear regression model [34] and the quadratic regression model [37] can be established, and their formulas are shown in Table 5.

The simulated results are shown in Table 6.

Based on the correlation coefficient and the relative error shown in Table 5 and Table 6, the GM(1,1) model, simple linear regression model, or quadratic regression model cannot be used for predicting the deaths of fatal accidents in metallurgical industry.

For this situation, the grey interval predicting method of the grey system theory is a suitable choice. The main procedures of grey interval predicting of fatal accidents are shown below.

The 1-AGO data of deaths due to fatal accidents is *X*^(1)^ = (16, 32, 51, 103, 150, 203, 288, 335, 370, 446, 491, 572, 619, 639, 663, 682, 709).

For the original data of deaths due to fatal accidents *X*^(0)^, $\sigma_{\max}=85$ and $\sigma_{\min}=16$.

The upper bound, lower bound and basic predictive functions of *x*^(1)^(*k*) are shown below.

(12) $$f_{u}\left(n+t\right)=709+85t$$

(13) $$f_{l}\left(n+t\right)=709+16t$$

(14) $$f_{b}\left(n+t\right)=\frac{\left(709+85t\right)+\left(709+16t\right)}{2}$$

Input *t* = 2 and *t* = 3 into Equations (12)–(14), the upper bound, lower bound and basic predictive values of 1-AGO data *x*^(1)^(19) and *x*^(1)^(20) can be achieved, with *f_u_*(*n* + 2) = 879, *f_l_*(*n* + 2) = 741, *f_b_*(*n* + 2) = 810, and *f_u_*(*n* + 3) = 964, *f_l_*(*n* + 3) = 757, and *f_b_*(*n* + 3) = 860.5. Then, input *f_u_*(*n* + 2) = 879 and *f_u_*(*n* + 3) = 964 into Equation (11), and the upper bound of original data can be achieved, with *x_u_*^(0)^(20) = 85. The lower bound and basic predictive values of the original data can be achieved in a similar way, with *x_l_*^(0)^(20) = 16 and *x_b_*^(0)^(20) = 50.5. The parameter *k* = 20 corresponds to the year 2020 in the time series. Therefore, the highest, lowest and basic predictive values of deaths due to fatal accidents in 2020 are 85, 16 and 50.5, respectively.

### 3.6. Severe Accident Prediction by the GM(1,1) Model

Catastrophe prediction is an outlier prediction in essence. For the threshold value of an outlier, it is generally determined subjectively by people.

Taking the severe accident as a catastrophe, first we predict the occurrence of a severe accident based on the simple linear regression model and quadratic regression model. Whether the severe accident occurs or not is a qualitative variable, and a corresponding value should be assigned to predict future characteristics. If a severe accident occurred, we assigned it 1, otherwise 0. Therefore, the data of severe accidents are *S* = (0, 0, 0, 1 0, 1, 1, 1, 0, 1, 1, 1, 0, 0, 1, 1). The time series of deaths due to fatal accidents *T* is taken as the independent variable and the data of severe accidents *S* is taken as the dependent variable. The formulas for predicting severe accidents based on the simple linear regression model and the quadratic regression model are shown in Table 7.

The simulated results of severe accidents based on the simple linear regression model and the quadratic regression model are shown in Table 8.

As shown in Table 7, the correlation coefficients of the simple linear regression model and the quadratic regression model are small, indicating that these two models are not suitable for predictions. In addition, every year in the future, severe accidents in the metallurgical industry will occur based on the simple linear regression model (Table 8). In contrast, severe accidents in metallurgical industry will not occur every year in the future based on the quadratic regression model (Table 8). Obviously, these two prediction results are not in accord with reality.

The GM(1,1) model of the grey system theory can be used for catastrophe prediction [42,43,44]. Taking a severe accident as a catastrophe, the time series of severe accidents is *T*^(0)^ = (4, 6, 7, 8, 10, 11, 12, 15, 16).

Taking *T*^(0)^ as the original data, the GM(1,1) model of severe accidents can be established based on Equations (1)–(6). The formula for predicting a severe accident is shown below.

$${\hat{x}}^{\left(0\right)}\left(k+1\right)=5.4174e^{0.1385t}.$$

The simulated results of severe accidents based on the GM(1,1) model are shown in Table 9.

The relative errors shown in Table 9 can pass the model test, therefore the GM(1,1) model of severe accidents can be used for predicting.

The next two values are 18.85 and 21.65 based on the formula for predicting severe accidents, indicating that in 2018.85 (2018 or 2019) and 2021.65 (2021 or 2022), there may be a severe accident in the metallurgical industry.

According to the relevant government websites in China, no severe accidents occurred in metallurgical enterprises in 2018 or 2019. However, a poisoning and apnoea accident caused by gas occurred at Shougang Group Shuicheng Steel Company in January 2018, which killed nine employees and injured another 2 [60]. This accident is extremely close to the severe accident category, indicating that the prediction method for severe accidents adopted in this paper is reasonable.

According to the prediction results, a severe accident will occur in metallurgical enterprises in 2021 or 2022, and corresponding countermeasures should be adopted to prevent these accidents.

### 3.7. Bow Tie Analysis of Fatal Accidents in the Metallurgical Industry

**Figure 7:**
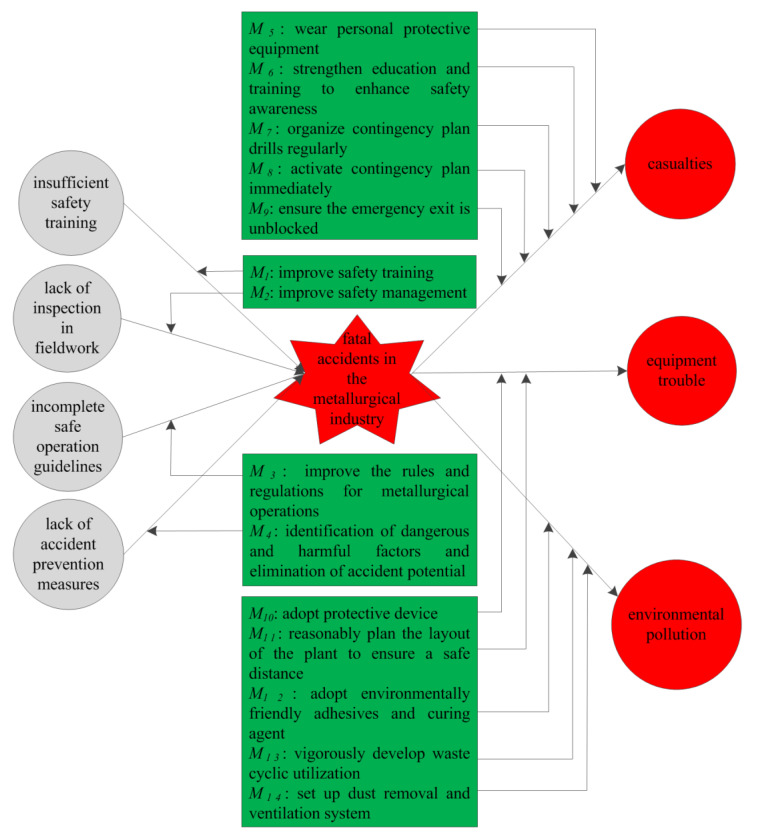
Bow tie analysis of fatal accidents in metallurgical enterprises.

In this paper, a bow tie analysis was carried out for fatal accidents in metallurgical enterprises, the causes and consequences of fatal accidents in metallurgical enterprises were identified and the corresponding safety measures to adopt for preventing such cases were identified (Figure 7).

Four causes that can lead to fatal accidents in metallurgical enterprises were listed on the left side of the bow tie, namely, insufficient safety training, lack of inspection in fieldwork, incomplete safe operation guidelines and lack of accident prevention measures, which belonged to fault tree analysis.

Three consequences of fatal accidents in metallurgical enterprises were set on the right side of the bow tie, namely, casualties, equipment trouble and environmental pollution, which belonged to event tree analysis.

Four corresponding preventive safety measures were listed on the left side and ten mitigative safety measures were listed on the right side. The precondition of mitigative safety measures is that an accident has occurred; its purpose is to reduce the loss from the accident. The best safety measures prevent accidents from happening and eliminate accidents from the start.

Through the detailed analysis of the bow tie model, the risk of fatal accidents in metallurgical enterprises can be reduced by adopting the corresponding safety measures. It should be mentioned that accidents are often caused by more than one factor. Therefore, to reduce the accident potential, multiple safety measures should be adopted to ensure safe production in metallurgical enterprises.

## 4. Discussion

As the original statistical data of fatal accidents in the metallurgical industry have irregular fluctuations, the traditional prediction methods, such as linear regression analysis [34], or quadratic regression analysis [37], cannot be used to predict their future characteristics. To overcome this issue, the grey system theory has been introduced to predict the future characteristics of fatal accidents in the metallurgical industry. The results of this study confirmed that the grey system theory can be used for interval and catastrophe predictions in the metallurgical industry.

Once the future characteristics of fatal accidents in the metallurgical industry have been determined, the corresponding safety measures should be adopted to prevent accidents. Different from fault tree analysis [45,46,47] or event tree analysis [48], the bow tie model integrates the basic causes, possible consequences, and corresponding safety measures of an accident in a transparent diagram [49,50,51]. In this study, the bow tie model was used to identify the causes and consequences of fatal accidents in the metallurgical industry; then, corresponding safety measures were adopted to reduce the risk of an accident occurring. Shin reported that the main cause of chemical accidents is safety work permit violations [23]. Additionally, the violation of a safety work permit is the main cause of accidents in the metallurgical industry.

According to the Heinrich accident-causing theory, approximately 80% of accidents are caused by unsafe human behavior [61]. Therefore, it is necessary to strengthen the safety training of employees in the metallurgical industry, standardize employee behavior, and improve employees’ professional skills. To do this, capital investment is required to improve the quality of safety training and enable employees to master the necessary safety skills. Second, different safety training plans are required for the operating features of different positions. Safety training must be understood by management and operable for employees. Third, evaluation is required to be sure that employees understand the safety training. The results from the evaluation can be applied to further improve the safety training content.

The object of safety management is not to deal with accidents but to manage the risk factors before an accident occurs. Safety management involves working to adopt appropriate technical and economical methods to effectively control the risk factors through hazard identification and safety assessments. Businesses should be aware of safety regulations and make these regulations part of standard operations. They must encourage employees to develop good safety behaviors. Safety management should focus on accident prevention. Regular safety inspections are needed to reduce the accident potential due to unsafe machinery conditions and unsafe employee behavior. Overall, efforts are needed to improve the safety awareness of employees and standardize operation procedures.

In many metallurgical enterprises, production accidents are caused by inadequate rules and regulations. Therefore, it is necessary to optimize labor organizations and improve the rules and regulations for operation. The optimization of labor organizations includes identifying different types of work and technology stages, describing the necessary preparatory and management work, setting specific operation teams, and putting limits on working time. The goal of labor organizations should be to establish a good labor productivity system to improve the relationships between employees, the work that needs to be done, and the instruments of labor. The rules and regulations for metallurgical companies should include a responsibility system, safe operation rules, and a management system. The rules and regulations should specify the safety responsibilities of each employee and standardize safe production behavior. Feasible emergency rescue plans should be designed and employees and rescuers should know this information to prevent the expansion of accidents caused by blind rescue efforts. It should be clear who is responsible for production safety and the implementation of the various rules and regulations to ensure safe production.

The identification of potentially dangerous and harmful factors is the foundation of risk management. Only after identifying these factors can the corresponding safety measures be constructed to control the accident potential. Risk analysis is required to assess the risk level and possible consequences of dangerous and harmful factors and to identify appropriate accident prevention measures. The metallurgical industry should strictly control and manage risks and design emergency rescue plans. Routine surveillance is also critical to control the identified risk factors.

To simplify the discussion, the initial condition of the first value of the 1-AGO data was chosen as only the first value of the original data in the GM(1,1) model. Future research should focus on the influence of the initial condition on the prediction result.

## 5. Conclusions

The purpose of this study was to establish a composite risk analysis method for fatal accidents in the metallurgical industry. The main conclusions can be summarized as follows.

Despite the serious consequences of fatal accidents in the metallurgical industry, few statistical analysis reports of fatal accidents in this industry exist. This study collected 152 fatal accidents in the Chinese metallurgical industry from 2001 to 2018, including 141 major accidents, 10 severe accidents, and 1 extraordinarily severe accident, together resulting in 731 deaths.

Most of the accidents in the traffic industry are object strikes, and approximately 50% of the accidents in the chemical industry are explosions. However, in the metallurgical industry, the greatest number of accidents are poisoning and asphyxiation accidents, which accounts for 40% of the total number of fatal accidents. The metallurgical industry performs many production processes, and gas may be used in many of these processes, which can lead to gas poisoning accidents. In addition, gas poisoning accidents in the metallurgical industry can also be caused by a gas leakage due to the corrosion of a pipeline or improper operation in a confined workspace.

The original statistical data of fatal accidents in the metallurgical industry have irregular fluctuations, so the traditional prediction methods, such as linear regression analysis or quadratic regression analysis, cannot be used to predict their future characteristics. To overcome this issue, the grey system theory is introduced to predict the future characteristics of fatal accidents in the metallurgical industry. The variation range of the fatal accidents was predicted based on the grey interval predicting method, and the GM(1,1) model was used for the catastrophe prediction. The results showed that severe accidents may occur in the metallurgical industry in 2021 or 2022, and corresponding countermeasures should be adopted to prevent these accidents.

Fault tree analysis can identify the causes of an accident but cannot perform a detailed analysis of the identified risk factors. Event tree analysis can perform a detailed analysis of the identified risk factors but cannot identify the causes of an accident. The bow tie model integrates the basic causes, possible consequences, and corresponding safety measures of an accident in a transparent diagram. In this study, the bow tie model was used to identify the causes and consequences of fatal accidents in the metallurgical industry; then, the corresponding safety measures were adopted to reduce the risk. The results showed that insufficient safety training, lack of inspection in fieldwork, incomplete safe operation guidelines and lack of accident prevention measures are the main causes that can lead to fatal accidents in the metallurgical industry. Casualties, equipment trouble and environmental pollution are the consequences of fatal accidents. The risk of fatal accidents in the metallurgical industry can be reduced by adopting the corresponding safety measures based on the bow tie model.

Although this study achieved several significant results, there are limitations to the proposed model. To simplify the discussion, the initial condition of the first value of the 1-AGO data was chosen as only the first value of the original data in the GM(1,1) model. Different GM(1,1) models can be established by choosing different initial conditions, which will have an influence on the prediction results. Future work should focus on the influence of the initial condition on the prediction results. In addition, this study only introduced the GM(1,1) model for catastrophe prediction in the metallurgical industry, and did not discuss the integration of the GM(1,1) model with other prediction techniques. Once the catastrophe data series were determined by the GM(1,1) model, linear or nonlinear regression models can also be used for prediction. Future studies should focus on the integration of the GM(1,1) model with other prediction techniques. Last but not least, bow tie model adopted in this study is a qualitative method, the severity and probability of an accident cannot be analyzed. A risk matrix is a structured risk management method, which can determine the risk level of an accident based on the probability and severity. To prevent the accident effectively, future research should focus on the feasibility analysis of combining risk matrix with bow tie model.

## Figures and Tables

**Table 1:** Production accident classification.

Accident Grade	Deaths	Serious Injuries	Property Loss (*Yuan*)
Ordinary	[0,3)	[0,10)	[0,10^7^)
Major	[3,10)	[10,50)	[10^7^,5 × 10^7^)
Severe	[10,30)	[50,100)	[5 × 10^7^,10^8^)

**Table 2:** Accuracy testing level.

Relative Error	0.01	0.05	0.10	0.20
Accuracy level	First level	Second level	Third level	Fourth level

**Table 3:** Severe and extraordinarily severe accidents in the Chinese metallurgical industry between 2001 and 2018.

Date	Company	Accident Type	Deaths	Injuries	Causes
8-April-2004	Zhongrun Iron and Steel Co. Ltd.	Electric shock	12	3	Employees accidentally touched 10,000 volt wires when moving a work shed.
8-November-2006	Yongqiang Roller Co. Ltd.	Thermal injury	12	15	Molten steel overflow due to a cover falling off a centrifugal casting machine.
18-April-2007	Qinghe Special Steel Co. Ltd.	Thermal injury	32	6	The steel ladle completely detached and molten steel was spilt and spread into the steelmaking workshop office.
19-August-2007	Weiqiao Pioneering Groups Co. Ltd.	Other explosion	20	55	Part of the aluminum discharge outlet was missing, allowing a large amount of high temperature aluminum liquid to flow and explode.
24-December-2008	Tangshan Ganglu Iron and Steel Co. Ltd.	Poisoning and asphyxiation	17	27	Gas leakage caused by a burst of the top explosion venting plate of the gravitational dust collector.
4-January-2010	Puyang Iron and Steel Co. Ltd.	Poisoning and asphyxiation	21	9	The converter gas recovery system was not sufficient, and the water seal was not constructed to the necessary design.
5-October-2011	Nanjing Iron and Steel Union Co. Ltd.	Thermal injury	12	1	The carbon brick inside the hearth had eroded and thinned, affecting the furnace casting when the strength monitoring and evaluation were insufficient. Additionally, the blowing operation caused an increase in the pressure in the furnace, which resulted in the molten iron breaking down the furnace wall.
20-February-2012	Anshan Iron and Steel Group	Other explosion	13	17	Water seepage of the pit caused excessive water accumulation at the bottom of the mold bed. A large amount of high-temperature molten steel was injected into the cavity for a short time, and the high energy was released as an explosion.
5-August-2012	Aluminum lock processing plant	Other explosion	13	14	Aluminum dust that accumulated during the polishing process detonated.
29-November-2015	Shandong Fulai Stainless Steel Co. Ltd.	Poisoning and asphyxiation	10	7	Defects in the drainer and gas transportation process allowed gas to leak.
22-Junuary-2016	Henan branch of China aluminum corporation	Significant fall	13	6	The bearing capacity of the subsider increased and was uneven due to improper construction that did not follow the design scheme. Employee operation in violation of rules.

**Table 4:** Accident types.

Accident Type	Description
Poisoning and asphyxiation	Poisoning is a human acute poisoning accident caused by exposure to a toxic substance; asphyxiation refers to what happens when working in a place that is not ventilated, employees may lose consciousness or die due to the lack of oxygen.
Other explosion	Explosion of furnace, iron or steel ladle.
Thermal injury	Burn caused by contact with flames, hot objects, or a strong acid or base splashing onto the body, or skin damage caused by radiation.
Collapse	Collapse of buildings, structures, stacked materials, or earth and stone.
Fall from a high place	Accidents caused by the difference in gravitational potential energy.
Object strike	Personal injury accidents caused by the inert force of an uncontrolled object.
Drowning	A large amount of water entering the lungs through the mouth or nose, blocking the respiratory tract, leading to acute hypoxia and suffocation.
Container explosion	Gas explosion caused by the rupture of the pressure vessel belongs to the physical explosion category.
Electric shock	Physical injury caused by electric current flowing through the human body.
Mechanical injury	Injury caused by twisting, smashing, bumping, or cutting by mechanical equipment and tools.
Fire disaster	Personal injury or death caused by fire.
Lifting injury	Accidents caused by the lifting operation.

**Table 5:** Prediction formulas of different models.

Model	Predicting Formula	Correlation Coefficient R
GM(1,1) model	${\hat{x}}^{\left(0\right)}\left(k+1\right)=-47e^{-0.0104t}$	Not applicable
Simple linear regression model	x=41.441+0.029t	0.007
Quadratic regression model	$x=-0.75t^{2}+13.608t-1.559$	0.728

**Table 6:** Simulated results of deaths caused by fatal accidents by different models.

Original Data	GM(1,1) Model		Simple Linear Regression Model		Quadratic Regression Model	
	Simulated Data	Relative Error	Simulated Data	Relative Error	Simulated Data	Relative Error
16	46.8043	192.5269%	41.4706	159.1912%	11.2952	72.7635%
19	46.3204	143.7914%	41.5000	159.375%	22.6404	45.4449%
52	45.8414	11.8434%	41.5294	118.5758%	32.4768	21.7981%
47	45.3675	3.4735%	41.5588	20.0792%	40.8044	1.8152%
53	44.8984	15.2861%	41.5882	11.5144%	47.6233	14.5115%
85	44.4341	47.7245%	41.6177	21.4761%	52.9334	27.1899%
47	43.9747	6.4368%	41.6471	51.0035%	56.7348	36.2276%
35	43.5200	24.3430%	41.6765	11.3267%	59.0274	41.6323%
76	43.0701	43.3289%	41.7059	19.1597%	59.8112	43.4118%
45	42.6247	5.2784%	41.7353	45.0851%	59.0862	41.5736%
81	42.1840	47.9210%	41.7647	7.18953%	56.8524	36.1255%
47	41.7478	11.1748%	41.7941	48.4023%	53.1099	27.0751%
20	41.3162	106.5809%	41.8235	11.0138%	47.8586	14.4299%
24	40.8890	70.3708%	41.8529	109.2647%	41.0986	1.80245%
19	40.4662	112.9801%	41.8824	74.50979%	32.8297	21.61443%
27	40.0478	48.3253%	41.9118	120.5882%	23.0521	44.99844%

**Table 7:** Formulas for predicting severe accidents.

Model	Prediction Formula	Correlation Coefficient R
Simple linear regression model	s=0.25+0.037t	0.342
Quadratic regression model	$s=-0.008t^{2}+0.165t-0.134$	0.446

**Table 8:** Simulated results of severe accidents.

Severe Accidents	Simple Linear Regression Model		Quadratic Regression Model	
	Simulated Data	Residual Error	Simulated Data	Residual Error
0	0.28676	−0.28676	0.02328	−0.28676
0	0.32353	−0.32353	0.16544	−0.32353
0	0.36029	−0.36029	0.29254	−0.36029
1	0.39706	0.60294	0.40459	0.60294
0	0.43382	−0.43382	0.50158	−0.43382
1	0.47059	0.52941	0.58351	0.52941
1	0.50735	0.49265	0.65039	0.49265
1	0.54412	0.45588	0.70221	0.45588
0	0.58088	−0.58088	0.73897	−0.58088
1	0.61765	0.38235	0.76068	0.38235
1	0.65441	0.34559	0.76733	0.34559
1	0.69118	0.30882	0.75893	0.30882
0	0.72794	−0.72794	0.73547	−0.72794
0	0.76471	−0.76471	0.69695	−0.76471
1	0.80147	0.19853	0.64338	0.19853
1	0.83824	0.16176	0.57475	0.16176

**Table 9:** Simulated results of severe accidents.

Original Data	Simulated Data	Residual Error	Relative Error
6	6.2231	−0.2231	3.7180%
7	7.1478	−0.1478	2.1118%
8	8.2100	−0.2100	2.6249%
10	9.4300	0.5700	5.7000%
11	10.8313	0.1687	1.5337%
12	12.4408	−0.4408	3.6736%
15	14.2895	0.7105	4.7364%
16	16.4130	−0.4130	2.5810%

## Supplementary Materials

The following are available online at https://www.mdpi.com/1660-4601/17/11/3790/s1, the data that supports this paper have been uploaded as electronic supplementary materials.

## References

[1] Ge J., Xu K., Zheng X., Yao X., Xu Q., Zhang B. (2019). The main challenges of safety science. Saf. Sci..

[2] Yan F., Xu K. (2018). A set pair analysis based layer of protection analysis and its application in quantitative risk assessment. J. Loss Prevent. Proc..

[3] Yao X., Zhou H., Xu K., Xu Q., Li L. (2020). Investigation on the fusion characterization and melting kinetics of ashes from co-firing of anthracite and pine sawdust. Renew. Energy.

[4] Xu Q., Xu K., Zhou F. (2020). Safety assessment of casting workshop by cloud model and cause and effect-LOPA to protect employee health. Int. J. Environ. Res. Public Health.

[5] Du Z., Lin B. (2018). Analysis of carbon emissions reduction of China’s metallurgical industry. J. Clean. Prod..

[6] Lin B., Xu M. (2018). Regional differences on CO_2_ emission efficiency in metallurgical industry of China. Energy Policy.

[7] Zhu X., Cao L., Liang Y. (2019). Spatial distribution and risk assessment of heavy metals inside and outside a typical lead-zinc mine in southeastern China. Environ. Sci. Pollut. Res..

[8] Brown P.P., Hardy N. (2019). Forecasting base metal prices with the Chilean exchange rate. Resour. Policy.

[9] Qi H., An H., Hao X., Zhong W., Zhang Y. (2014). Analyzing the international exergy flow network of ferrous metal ores. PLoS ONE.

[10] Gu J., Yao J., Jordan G., Roha B., Min N., Lu C. (2020). Arundo donax L. stem-derived biochar increases As and Sb toxicities from nonferrous metal mine tailings. Environ. Sci. Pollut. Res..

[11] Liu J., Yao J., Duran R., Mihucz V.G., Hudson-Edwards K.A. (2019). Bacterial shifts during in-situ mineralization bio-treatment to nonferrous metal(loid) tailings. Environ. Pollut..

[12] (2019). World Steel Association. https://www.worldsteel.org/.

[13] Dell T., Berkhout J. (1998). Injuries at a Metal Foundry as a Function of Job Classification, Length of Employment and Drug Screening. J. Saf. Res..

[14] Xu Q., Xu K., Li L., Xu X., Yao X. (2019). Energy release and countermeasures for sand casting explosion accidents. Hum. Ecol. Risk Assess..

[15] Lind S., Kivisto-Rahnasto J. (2008). Utilization of external accident information in companies’ safety promotion—Case: Finnish metal and transportation industry. Saf. Sci..

[16] State Administration of Work Safety (2018). Online Accident Inquiry System. http://media.chinasafety.gov.cn:8090/iSystem/shigumain.jsp.

[17] Baker A., Heiler K., Ferguson S. (2003). The impact of roster changes on absenteeism and incident frequency in an Australian coal mine. Occup. Environ. Med..

[18] Zhang J., Cliff D., Xu K., You G. (2018). Focusing on the patterns and characteristics of extraordinarily severe gas explosion accidents in Chinese coal mines. Process Saf. Environ. Prot..

[19] Zhang J., Xu K., Reniers G., You G. (2020). Statistical analysis the characteristics of extraordinarily severe coal mine accidents (ESCMAs) in China from 1950 to 2018. Process Saf. Environ. Prot..

[20] Sze N.N., Wong S.C. (2007). Diagnostic analysis of the logistic model for pedestrian injury severity in traffic crashes. Accid. Anal. Prev..

[21] Huang W., Liu Y., Zhang Y., Zhang R., Xu M., De Dieu G.J., Antwi E., Shuai B. (2020). Fault Tree and Fuzzy D-S Evidential Reasoning combined approach: An application in railway dangerous goods transportation system accident analysis. Inf. Sci..

[22] Ma C., Zhou J., Yang D. (2020). Causation Analysis of Hazardous Material Road Transportation Accidents Based on the Ordered Logit Regression Model. Int. J. Environ. Res. Public Health.

[23] Shin I.J. (2013). Major industrial accidents in Korea: The characteristics and implication of statistics 1996–2011. Process Saf. Prog..

[24] Jung S., Woo J., Kang C. (2020). Analysis of severe industrial accidents caused by hazardous chemicals in South Korea from January 2008 to June 2018. Saf. Sci..

[25] Du L., Feng Y., Tang L., Lu W., Kang W. (2020). Time dynamics of emergency response network for hazardous chemical accidents: A case study in China. J. Clean. Prod..

[26] Li C., Qin J., Li J., Hou Q. (2016). The accident early warning system for iron and steel enterprises based on combination weighting and grey prediction model GM(1,1). Saf. Sci..

[27] Berhan E. (2020). Prevalence of occupational accident and injuries and their associated factors in iron, steel and metal manufacturing industries in Addis Ababa. Cogent Eng..

[28] Zhang L., Wang L., Zheng Y., Wang K., Zhang X., Zheng Y. (2017). Time prediction models for echinococcosis based on gray system theory and epidemic dynamics. Int. J. Environ. Res. Public Health.

[29] Wang S., Wang P., Zhang Y. (2020). A prediction method for urban heat supply based on grey system theory. Sustain. Cities Soc..

[30] Xu Q., Xu K. (2019). Quality evaluation of Chinese red wine based on cloud model. J. Food Biochem..

[31] Islam B., Baharudin Z., Nallagownden P. (2017). Development of chaotically improved meta-heuristics and modified BP neural network-based model for electrical energy demand prediction in smart grid. Neural Comput. Applic..

[32] Xu Q., Xu K., Li L., Yao X. (2019). Optimization of sand casting performance parameters and missing data prediction. R. Soc. Open Sci..

[33] Chang Y., Yue J., Guo R., Liu W., Li L. (2020). Penetration quality prediction of asymmetrical fillet root welding based on optimized BP neural network. J. Manuf. Process..

[34] Guo X., Song L., Fang Y., Zhu L. (2019). Model checking for general linear regression with nonignorable missing response. Comput. Stat. Data Anal..

[35] Stifft F., Vandermeer F., Neef C., Van Kuijk S., Christiaans M.H.L. (2020). A limited sampling strategy to estimate exposure of once-daily modified release tacrolimus in renal transplant recipients using linear regression analysis and comparison with Bayesian population pharmacokinetics in different cohorts. Eur. J. Clin. Pharmacol..

[36] Luo S., Tian J., Liu Z., Lu Q., Zhong K., Yang X. (2020). Rapid determination of styrene-butadiene-styrene (SBS) content in modified asphalt based on Fourier transform infrared (FTIR) spectrometer and linear regression analysis. Measurement.

[37] Ghanbarzadeh Lak M., Sabour M.R., Amiri A., Rabbani O. (2012). Application of quadratic regression model for Fenton treatment of municipal landfill leachate. Waste Manag..

[38] Xu X., Qiu M., Liu J., Niu Z., Wu X. (2019). Prediction of maximal water bursting discharge from coal seam floor based on multiple nonlinear regression analysis. Arab. J. Geosci..

[39] Liu J., Xiao X., Guo J., Mao S. (2014). Error and its upper bound estimation between the solutions of GM(1,1) grey forecasting models. Appl. Math. Comput..

[40] Xiong P., He Z., Chen S., Peng M. (2020). A novel GM(1,N) model based on interval gray number and its application to research on smog pollution. Kybernetes.

[41] Ye J., Dang Y., Ding S., Yang Y. (2019). A novel energy consumption forecasting model combining an optimized DGM (1,1) model with interval grey numbers. J. Clean. Prod..

[42] Miao C., Ding M. (2017). Analysis of influence of natural disaster on the economy and prediction of recovery time based on grey forecasting-difference comparison model: A case study in the upper Min River. Nat. Hazards.

[43] Bai H., Yu G. (2016). A Weibo-based approach to disaster informatics: Incidents monitor in post-disaster situation via Weibo text negative sentiment analysis. Nat. Hazards.

[44] Zhou Q., Hu Q., Ai M., Xiong C., Jin H. (2020). An improved GM(1,3) model combining terrain factors and neural network error correction for urban land subsidence prediction. Geomat. Nat. Hazards Risk.

[45] Bas H., Elevli S., Yapici F. (2019). Fault Tree Analysis for Fused Filament Fabrication Type Three-Dimensional Printers. J. Fail. Anal. Prev..

[46] Hu L., Kang R., Pan X., Zuo D. (2020). Risk assessment of uncertain random system-Level-1 and level-2 joint propagation of uncertainty and probability in fault tree analysis. Reliab. Eng. Syst. Saf..

[47] Yazdi M., Korhan O., Daneshvar S. (2020). Application of fuzzy fault tree analysis based on modified fuzzy AHP and fuzzy TOPSIS for fire and explosion in the process industry. Int. J. Occup. Saf. Ergon..

[48] Rahman S., Karanki D.R., Epiney A., Wicaksono D., Zerkak O., Dang V.N. (2018). Deterministic sampling for propagating epistemic and aleatory uncertainty in dynamic event tree analysis. Reliab. Eng. Syst. Saf..

[49] Xu Q., Xu K., Li L., Yao X. (2018). Safety assessment of petrochemical enterprise using the cloud model, PHA–LOPA and the bow-tie model. R. Soc. Open Sci..

[50] Xu Q., Xu K. (2018). Mine safety assessment using gray relational analysis and bow tie model. PLoS ONE.

[51] Xu Q., Xu K. (2018). Risk assessment of rail haulage accidents in inclined tunnels with Bayesian network and bow-tie model. Curr. Sci..

[52] Rajesh R., Rajendran C. (2019). Grey-and rough-set-based seasonal disaster predictions: An analysis of flood data in India. Nat. Hazards.

[53] Fu Y., Yao J., Zhao H., Zhao G., Wan Z. (2018). Forecast for artificial muscle tremor behavior based on dynamic additional grey catastrophe prediction. Appl. Sci..

[54] Shen X., Ou L., Chen X., Zhang X., Tan X. (2013). The application of the grey disaster model to forecast epidemic peaks of typhoid and paratyphoid fever in China. PLoS ONE.

[55] Huang Y., Lin C. (2011). Developing an interval forecasting method to predict undulated demand. Qual. Quant..

[56] Chen Y., Liu H., Hsieh H. (2019). Time series interval forecast using GM(1,1) and NGBM(1,1) models. Soft Comput..

[57] Li J., Xu K., Fan B., Geng L. (2020). Risk assessment of oxygen lance burning loss using bow-tie analysis based on fuzzy theory. Math. Probl. Eng..

[58] Cui Y., Tian S.S., Qiao N., Wang C., Wang T., Huang J.J., Sun C.-M., Liang J., Liu X.-M. (2015). Associations of individual-related and job-related risk factors with nonfatal occupational injury in the coal workers of Shanxi province: A cross-sectional study. PLoS ONE.

[59] He G., Zhang L., Lu Y., Mol A.P. (2011). Managing major chemical accidents in China: Towards effective risk information. J. Hazard Mater..

[60] Ministry of Emergency Management of the People’s Republic of China (2018). Notification. https://www.mem.gov.cn/gk/gwgg/agwzlfl/tb_01/201802/t20180206_239702.shtml.

[61] Low B.K.L., Man S.S., Chan A.H.S. (2018). The risk-taking propensity of construction workers-an application of quasi-expert interview. Int. J. Environ. Res. Public Health.

