# Supplementary material for: Statistical Analysis and Prediction of Fatal Accidents in the Metallurgical Industry in China
Source: Int J Environ Res Public Health. 2020 May 27;17(11):3790. doi: 10.3390/ijerph17113790 (PMC7312879; doi:10.3390/ijerph17113790)
Supplement: Supplementary file 1 [file ijerph-17-03790-s001.pdf]

Date(year-month-day)	Company
2018/5/17	Nanyang hanye special steel co. LTD
2018/4/3	Yuncheng yunhai aluminum co. LTD
2018/2/5	Shaoguan Songshan Iron and Steel Group co. LTD
2018/1/31	Shougang Group shuicheng steel co. LTD
2017/12/14	Beiman special steel co. LTD
2017/9/20	Duopu alloy co. LTD
2017/8/3	Desheng steel co. LTD
2017/5/14	Beiman special steel co. LTD
2017/3/20	Hongxin metal recycling co. LTD
2017/3/20	Henan yuguang gold and lead co. LTD
2017/3/10	Guangxi huayin aluminum co. LTD
2017/3/6	Luoyang iron and steel co. LTD
2016/10/16	Xinjiang kunyu iron and steel co. LTD
2016/6/22	Henan branch of China aluminum corporation
2016/4/1	Linyi sande special steel co. LTD
2015/11/29	Shandong Fulai Stainless Steel Co. Ltd.
2015/9/2	Beijing Shougang Co., Ltd
2015/8/31	Huaxinyuan iron and steel co.
2015/4/2	Henan Zhongyuan Special Steel Co.,Ltd.
2014/12/8	Jiyuan iron and steel co. LTD
2014/8/6	Shanxi huaze of China aluminum corporation
2014/7/2	Fengzhen city fengye ferro-alloy co. LTD
2014/3/28	Guangxi shenglong metallurgy co. LTD
2014/3/23	Yukun iron and steel group co. LTD
2014/1/7	The second plant of Taiyuan iron and steel (group) co. LT
2013/12/5	Jinxu steel structure co. LTD
2013/11/21	North steel group jinding heavy industry co. LTD
2013/11/11	Xinjiang bayi steel structure co. LTD
2013/9/29	Kunming iron and steel co. LTD
2013/8/25	Xijin mining and manganese alloy co.
2013/7/30	Wenan new steel company
2013/7/22	Yunnan yuxi xianfu iron and steel (group) co. LTD
2013/5/4	Iron works of Taiyuan iron and steel (group) co. LTD
2013/4/17	Julong metal materials co. LTD
2013/4/1	Xinyu xingang company
2013/1/16	Panchenggang construction company
2012/12/20	Xinyu xingang company
2012/12/6	Suzhou huasheng bondi copper plated steel band co. LTD
2012/8/15	Changfeng steel co. LTD
2012/8/5	Aluminum lock processing plant
2012/7/31	West steel group
2012/7/8	Hunan valin iron and steel co. LTD

2012/6/25	Benxi iron and steel (group) co.
2012/6/18	Guangxi sha gang manganese co. LTD
2012/5/25	Tonggang group
2012/5/14	Guangdong shaoguan iron and steel group co.
2012/4/26	Hebei puyang iron and steel co. LTD
2012/3/20	Jiugang group iron works
2012/2/23	Shanghai baosteel group meishan iron and steel co.
2012/2/20	Anshan Iron and Steel Group
2012/2/11	Guizhou guanglv aluminum industry co. LTD
2012/2/5	Dazhou steel group co. LTD
2012/1/26	Zijin copper co. LTD
2011/10/14	Shandong shiheng special steel group co. LTD
2011/10/5	Nanjing Iron and Steel Union Co. Ltd.
2011/8/14	Neimenggu baogang steel works
2011/6/11	Changzhou zhongyue foundry plant
2011/5/4	Anshan iron & steel co. LTD
2011/4/30	Weifang special steel group co. LTD
2011/4/17	Jinan jianchang machinery factory
2011/4/17	Guizhou hezhang dianqian honest smelting co. LTD
2011/1/13	Jinan second machine group co. LTD
2011/1/11	Tonghai xinguang industry and trade co. LTD
2010/12/17	Jiangyin zhaohong machinery co. LTD
2010/12/8	Furen iron and steel co. LTD
2010/10/5	Shanxi tisco stainless steel co. LTD
2010/9/29	Henan xichuan aluminum (group) co. LTD
2010/9/26	Liaoning anshan iron and steel construction group
2010/9/23	Yunfeng aluminum co. LTD
2010/9/9	Wuhan iron and steel general plant sintering branch factor
2010/8/28	Tangshan iron and steel company
2010/6/27	Luliang central iron and steel group co.
2010/5/29	Sanming steel plant metallurgical construction company
2010/5/17	Houma copper smelter in northern China
2010/1/12	Jinshan iron and steel co. LTD
2010/1/4	Liaoning dalian special steel co. LTD
2010/1/4	Puyang Iron and Steel Co. Ltd.
2009/12/21	China railway 14th bureau jinan iron and steel group
2009/12/6	Xinyu Iron And Steel Co.Ltd
2009/12/3	Guangdong west foundry
2009/9/19	Xiangfen qiangsheng iron alloy plant
2009/8/24	Zhiqiang iron and steel co.
2009/7/15	Wuxi xinsanzhou special steel co. LTD
2009/4/2	Laigang group yinshan section steel co. LTD
2009/3/21	Shougang jingtang iron and steel co.

2009/1/21	Shaanxi hanzhong iron and steel group co. LTD
2009/1/17	Qingdao huaye casting co. LTD
2008/12/24	Tangshan Ganglu Iron and Steel Co. Ltd.
2008/12/16	Chongqing taizheng mineral resources development co. L
2008/11/15	Zhongyu iron and steel co. LTD
2008/10/18	Wuhan iron and steel corporation egang plant
2008/9/24	Double brand aluminum industry co. LTD
2008/9/5	Wenan xingang iron and steel co. LTD
2008/3/14	Yongjian casting co. LTD
2007/9/29	Kunming iron and steel group co. LTD
2007/9/9	Huaniushan group co. LTD
2007/8/19	Weiqiao Pioneering Groups Co. Ltd.
2007/7/20	Jiangsu shagang group huaigang special steel co. LTD
2007/6/6	Qianan liangangl xinda steel co. LTD
2007/4/27	Houying group haicheng steel co. LTD
2007/4/18	Qinghe Special Steel Co. Ltd.
2007/3/19	Smelting branch of hangzhou iron and steel group co. LT
2007/3/8	Maanshan steel works
2007/1/30	Xichuan xiwan crystal silicon plant
2006/12/26	United steel yanshan iron and steel co.
2006/11/13	Zhongzhou branch of China aluminum corporation
2006/11/8	Yongqiang Roller Co. Ltd.
2006/10/8	HANGZHOU IRON AND STEEL GROUP CO.
2006/7/21	Luohe iron and steel co. LTD
2006/7/7	WISCO Ironworks
2006/6/11	Jinan iron and steel group corporation
2006/3/30	Guofeng iron and steel co. LTD
2006/1/10	Chenggang iron and steel co.
2006/1/6	Shuicheng steel (group) co. LTD
2005/11/5	Daan iron and steel co.
2005/10/26	Shougang power plant
2005/10/8	Handan xinxing cast pipe co. LTD
2005/9/27	Ling hai electric steel co. LTD
2005/8/18	Longmen iron and steel group corporation
2005/8/12	Qiaoxi foreign trade foundry
2005/7/7	LAIWU STEEL GROUP co., ltd
2005/7/5	Handan Iron and Steel Group Company
2005/6/23	Ankang Guangda Ferroalloy Factory
2005/5/20	Jinan Iron and Steel Group gas power plant
2005/4/14	Jinggang iron and Steel Co., Ltd.
2005/3/2	New fushun special steel shares Co., Ltd.
2005/2/19	Hebei jinxi iron and steel co. LTD
2004/11/28	Hanzhong iron and steel co. LTD

2004/11/13	Hebei jinxi iron and steel co. LTD
2004/9/27	Bazhou xinli steel co. LTD
2004/8/30	Shanxi branch of China aluminum corporation
2004/6/11	Jinsheng steel co. LTD
2004/5/19	Longmen iron and steel (group) co.
2004/5/17	Dongxin foundry
2004/4/8	Zhongrun Iron and Steel Co. Ltd.
2004/4/5	Shuicheng steel (group) co. LTD
2004/1/12	Hubei huang metallurgical steel group
2003/11/24	Yuzhong iron and steel co., LTD
2003/9/15	Longmen iron and steel (group) co.
2003/9/9	Zhengyang casting steel factory
2003/6/29	Shuicheng steel (group) co. LTD
2003/5/25	Panzhihua Iron and Steel Group Co. , Ltd
2003/3/22	Baotou Iron and Steel Company
2002/11/19	Baotou Iron and Steel Company
2002/10/29	Zunyi xiangjiang steel plant
2002/9/14	Baotou steel comprehensive enterprise group co. LTD
2002/2/8	Benxi iron and steel co.
2002/1/31	Beigang group beiying steelmaking co. LTD
2001/12/28	Jinzhou ferroalloy (group) co. LTD
2001/10/20	Industrial sewing machine factory
2001/10/16	Shanghai meishan steel corporation
2001/10/13	Chengde steel works

Note: the blank of injury indicates that there is no injury record in the accident an

Accident type	Deathes	Injuries
Other explosion	2	13
Other explosion	3	3
Poisoning and apnea	8	10
Poisoning and apnea	9	2
Container explosion	3	
Poisoning and apnea	4	
Poisoning and apnea	3	6
Other explosion	3	1
Thermal injury	5	2
Other explosion	3	
Poisoning and apnea	3	
Poisoning and apnea	3	
Poisoning and apnea	3	
Fall from a high place	13	6
Thermal injury	3	
Poisoning and apnea	10	7
Drowning	7	
Poisoning and apnea	4	4
Poisoning and apnea	3	3
Drowning	3	
Other explosion	4	
Collapse	4	
Poisoning and apnea	3	
Poisoning and apnea	2	17
Poisoning and apnea	4	2
Collapse	3	2
Mechanical injury	4	3
Other explosion	6	6
Poisoning and apnea	3	
Poisoning and apnea	7	4
Other explosion	7	1
Other explosion	3	2
Poisoning and apnea	3	1
Thermal injury	3	1
Other explosion	4	26
Poisoning and apnea	4	2
Fall from a high place	3	5
Poisoning and apnea	3	2
Container explosion	3	
Other explosion	13	14
Collapse	3	5
Poisoning and apnea	3	3

Fall from a high place	3	
Collapse	3	5
Fall from a high place	3	2
Other explosion	9	6
Poisoning and apnea	3	5
Object strike	3	
Poisoning and apnea	7	7
Other explosion	13	17
Object strike	3	1
Thermal injury	3	
Poisoning and apnea	3	1
Collapse	3	1
Thermal injury	12	1
Poisoning and apnea	4	
Poisoning and apnea	6	1
Poisoning and apnea	3	
Poisoning and apnea	3	17
Object strike	3	
Poisoning and apnea	4	
Fall from a high place	4	3
Collapse	3	
Object strike	3	
Lifting injury	3	5
Poisoning and apnea	3	
Collapse	5	11
Container explosion	3	1
Collapse	9	4
Mechanical injury	5	
Poisoning and apnea	3	
Collapse	4	1
Poisoning and apnea	3	
Other explosion	3	
Thermal injury	3	
Poisoning and apnea	8	
Poisoning and apnea	21	9
Fall from a high place	3	
Poisoning and apnea	4	1
Other explosion	3	1
Poisoning and apnea	4	
Poisoning and apnea	3	3
Poisoning and apnea	3	8
Fall from a high place	3	2
Poisoning and apnea	5	

Fall from a high place	3	1
Thermal injury	4	1
Poisoning and apnea	17	27
Collapse	7	11
Poisoning and apnea	3	3
Poisoning and apnea	3	11
Other explosion	4	3
Poisoning and apnea	7	
Collapse	6	2
Poisoning and apnea	3	1
Other explosion	8	10
Other explosion	20	55
Container explosion	4	1
Poisoning and apnea	3	12
Poisoning and apnea	4	
Thermal injury	32	6
Object strike	4	
Fall from a high place	3	
Thermal injury	4	4
Fall from a high place	4	1
Collapse	3	
Thermal injury	12	15
Poisoning and apnea	3	
Poisoning and apnea	3	
Collapse	5	2
Poisoning and apnea	6	
Other explosion	6	6
Poisoning and apnea	4	6
Poisoning and apnea	7	21
Poisoning and apnea	5	1
Poisoning and apnea	9	
Collapse	3	
Other explosion	3	13
Poisoning and apnea	3	5
Collapse	3	2
Fall from a high place	3	1
Poisoning and apnea	1	12
Thermal injury	3	
Poisoning and apnea	3	19
Container explosion	3	4
Fire disaster	5	1
Object strike	3	1
Collapse	7	

Thermal injury	3	3
Poisoning and apnea	5	
Poisoning and apnea	3	
Other explosion	5	12
Other explosion	3	1
Collapse	3	5
Electric shock	12	3
Object strike	8	12
Poisoning and apnea	3	
Fall from a high place	3	
Drowning	5	4
Fire disaster	3	7
Poisoning and apnea	3	29
Collapse	3	3
Object strike	2	11
Poisoning and apnea	3	
Thermal injury	3	5
Poisoning and apnea	3	
Poisoning and apnea	4	
Thermal injury	3	
Other explosion	4	6
Other explosion	3	1
Fall from a high place	6	3
Thermal injury	3	

alysis report.
